# Structural Characterization and Anti‐Alzheimer's Disease Effect of Polysaccharides From Stellariae Radix

**DOI:** 10.1002/fsn3.71604

**Published:** 2026-03-11

**Authors:** Meixia Sun, Jiaxin He, Huan Song, Zhi Ma, Xilian Zhang, Juan Li, Yao Yao

**Affiliations:** ^1^ School of Pharmacy Ningxia Medical University Yinchuan People's Republic of China; ^2^ Ningxia Engineering and Technology Research Center for Modernization of Characteristic Chinese Medicine, and Key Laboratory of Ningxia Ethnomedicine Modernization, Ministry of Education Ningxia Medical University Yinchuan People's Republic of China; ^3^ School of Basic Medical Sciences Ningxia Medical University Yinchuan People's Republic of China

**Keywords:** Alzheimer's disease, antioxidant, polysaccharide, Stellariae Radix, structural characterization

## Abstract

Stellariae Radix, a frequently employed traditional Chinese medicine, originates from the dried roots of *Stellaria dichotoma* L. *var. lanceolata* Bge. To elucidate the structural characteristics and anti‐Alzheimer's disease (AD) efficacy of *S. dichotoma* polysaccharides (SDP), SDP was extracted and comprehensively characterized using ultraviolet–visible spectroscopy (UV–Vis), Fourier‐transform infrared spectroscopy (FT‐IR), nuclear magnetic resonance (NMR) spectroscopy and high‐performance liquid chromatography (HPLC). The results revealed that SDP is composed of galactose, glucose, arabinose, galacturonic acid, mannose, and rhamnose at a molar ratio of 5.561:2.224:0.802:0.616:0.613:0.184. In vitro experiments demonstrated that SDP exhibited potent scavenging activities against ABTS, DPPH, and hydroxyl radicals in a dose‐dependent manner, with the average scavenging rates reaching 99.07%, 89.44% and 56.43% respectively at the concentration of 5 mg/mL. In a C57BL/6J mouse model of AD, administration of SDP (50–200 mg/kg) significantly ameliorated cognitive dysfunction, increased the hippocampal levels of superoxide dismutase (SOD) and glutathione peroxidase (GSH‐Px), decreased the malondialdehyde (MDA) content, and regulated the expression of oxidative stress‐related proteins, including Nrf2, Keap1 and NQO1. These findings indicate that SDP possesses antioxidant and neuroprotective properties, suggesting its potential therapeutic value for the treatment of AD.

## Graphical abstract

*Stellaria dichotoma* polysaccharides (SDP) was extracted from Stellariae Radix and comprehensively characterized using UV–Vis, FT‐IR, NMR spectroscopy and HPLC analysis. The results revealed that SDP is composed of galactose, glucose, arabinose, galacturonic acid, mannose, and rhamnose. In vitro experiments demonstrated that SDP exhibited potent scavenging activities against ABTS, DPPH, and hydroxyl radicals in a dose‐dependent manner. In AD mice model, SDP significantly ameliorated cognitive dysfunction, increased the hippocampal levels of SOD and GSH‐Px, decreased the MDA content, and regulated the expression of oxidative stress‐related proteins, including Nrf2, Keap1 and NQO1.

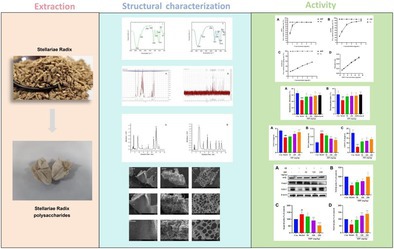

**Abbreviations**
AD: Alzheimer's disease
GSH‐Px: glutathione peroxidase
HPLC: high‐performance liquid chromatography
Keap1: Kelch‐like ECH‐associated protein 1
MDA: malondialdehyde
NMR: nuclear magnetic resonance
NQO1: quinoneoxidoreductase1
Nrf2: nuclear factor erythroid 2‐related factor 2
SEM: scanning electron microscopy
SOD: superoxide dismutase
UV: ultraviolet spectrum

## 1 Introduction

*Stellaria dichotoma* L. *var. lanceolata* Bge. is a perennial herb which belongs to the *Caryophyllaceae* family. Its dried roots have been used in traditional Chinese medicine as Stellariae Radix (Yinchaihu). Stellariae Radix is commonly used to treat symptoms such as fever due to yin deficiency, consumptive fever, and infantile malnutrition fever. Modern phytochemical studies have revealed that Stellariae Radix contains various bioactive constituents, including alkaloids (Zhou et al. 2023), cyclic peptides, sterols, phenolic acids, volatile oils, and polysaccharides (Ma et al. 2023), which have shown extensive applications in pharmaceuticals, healthcare products, and the food industry. However, although significant progress has been achieved in the chemical investigation of Stellariae Radix, research that focuses on its polysaccharide components remains relatively limited. As one of its key bioactive constituents, SDP holds significant potential for further exploration and development.

Polysaccharides are macromolecular carbohydrates formed by the linkage of multiple monosaccharide units through glycosidic bonds (Ji et al. 2023), and they are ubiquitously present in plants, animals, and microorganisms (Kakar and Chhalgari 2020). These biomolecules serve as essential components of cell membrane receptors and participate in various essential biological processes, including cell recognition, signal transduction, and immune regulation (Balavigneswaran et al. 2013). Apart from their roles as energy storage molecules and structural components, polysaccharides exhibit significant biological activities. In recent years, plant‐derived polysaccharides have attracted increasing attention due to their unique bioactivities and functional properties. Studies have demonstrated that many plant polysaccharides possess excellent emulsifying and thickening properties, making them valuable stabilizers in food and cosmetic formulation (Shao et al. 2020). Moreover, plant polysaccharides have been well‐documented to exhibit diverse biological activities such as scavenging free radicals and mitigating oxidative stress, thus protecting cells from damage (Huang et al. 2017), enhancing immune function through the activation of macrophages, T cells, and B cells (Hu et al. 2021), inhibiting tumor cell proliferation and inducing apoptosis (Lai et al. 2024), regulating inflammatory factor expression to reduce inflammatory responses (Wei et al. 2021), as well as acting as prebiotics to promote beneficial bacterial growth and restore intestinal microecological balance (Xue et al. 2024). These plant polysaccharides exhibit broad prospects for applications in functional foods and healthcare products.

Based on previous evidence, we hypothesized that SDP, as a bioactive polysaccharide from Stellariae Radix, possesses potent antioxidant activity. We also proposed that SDP could alleviate oxidative stress in the brain, thereby ameliorating cognitive impairment in Alzheimer's disease (AD) mice and exerting neuroprotective effects. In this study, SDP was extracted and comprehensively characterized using ultraviolet–visible spectroscopy (UV–Vis), Fourier‐transform infrared spectroscopy (FT‐IR), nuclear magnetic resonance (NMR) spectroscopy, and high‐performance liquid chromatography (HPLC) analysis to elucidate its structural characteristics. Its anti‐oxidative stress and anti‐AD efficacy was further investigated in vitro and in an AD mice model.

## 2 Materials and Methods

### 2.1 Preparation of SDP

Stellariae Radix was purchased from Ningxia Mingde Pharmaceutical Co. Ltd. (Guyuan, China). The dried roots were ground into fine powder by using a traditional Chinese medicine grinder and sifted through a 40‐mesh sieve. The powder was extracted three times at 100°C with a solid‐to‐liquid ratio of 1:15 for 90 min each time. The combined extracts were concentrated using a rotary evaporator and precipitated overnight at 4°C with 95% ethanol to obtain crude SDP. After filtration, the crude SDP was deproteinized using the Sevage method. The Sevage reagent was prepared by mixing chloroform and n‐butanol at a volume ratio of 4:1. The deproteinization operation was performed as follows: the crude SDP solution was mixed with the Sevage reagent at a volume ratio of 3:1, shaken vigorously for 30 min, and centrifuged at 8000 r/min for 10 min. The upper aqueous phase containing polysaccharides was collected, and this process was repeated 3 times until no white flocculent protein precipitate appeared at the interface.

### 2.2 Determination of Total Saccharide Content

The total saccharide content of the purified SDP was determined using the phenol‐sulfuric acid method (Yue et al. 2022) and found to be 86.37%. The specific steps of the phenol‐sulfuric acid method were as follows: Prepare standard glucose solutions with concentrations of 0, 10, 20, 30, 40, 50, and 60 μg/mL. Pipette 1 mL of standard glucose solution or sample solution into a test tube, add 1 mL of 5% phenol solution and mix well, then quickly add 5 mL of concentrated sulfuric acid and shake vigorously. Place the test tube in a water bath at 60°C for 15 min, then cool to room temperature. Measure the absorbance value at 485 nm using a UV–visible spectrophotometer. Draw a standard curve with glucose concentration as the abscissa and absorbance value as the ordinate, and calculate the total saccharide content of the sample according to the standard curve.

### 2.3 Ultraviolet (UV) Spectrum Analysis

An appropriate amount of purified SDP was weighed and analyzed using a UV–visible spectrophotometer. Scans were conducted between 200 nm and 600 nm, with specific detections at 260 nm (nucleic acids) and 280 nm (proteins) to assess the presence of impurities.

### 2.4 Infrared Spectrum Analysis

Approximately 1‐2 mg of SDP and 200 mg of potassium bromide powder (ratio 1:100) were rapidly weighed to prevent moisture absorption. The mixture was thoroughly ground in an agate mortar cleaned with alcohol and pressed into a transparent, crack‐free pellet. The pellet was scanned within the range of 400–4000 cm^−1^ by an infrared spectrometer to analyze the organic functional groups of the polysaccharide.

### 2.5 Scanning Electron Microscopy (SEM)

A small portion of dried SDP sample was dispersed on double‐sided adhesive fixed onto a platform. Excess sample powder was removed using a hair dryer, ensuring a thin layer of SDP on the adhesive. The sample was then gold‐coated in a vacuum coater and placed in the SEM chamber to observe the morphology of the sample in its solid state.

### 2.6 Nuclear Magnetic Resonance (NMR) Analysis

The SDP powder was dissolved in 0.8 mL of D_2_O, fully dissolved, and centrifuged before being transferred to an NMR tube. ^1^H NMR and ^13^C NMR spectra were recorded using an NMR instrument.

### 2.7 Determination of Monosaccharide Composition

The monosaccharides in SDP were identified using PMP‐HPLC analysis. Standards, including mannose, rhamnose, galacturonic acid, glucose, galactose, and arabinose, were prepared at a concentration of 2 mg/mL in deionized water. SDP samples were hydrolyzed in trifluoroacetic acid at 110°C for 2 h, concentrated using a rotary evaporator, and derivatized with PMP‐methanol. After purification, HPLC analysis was performed using a YMC‐PACK Pro C18/S‐5 μm column (4.6 mm × 250 mm) with acetonitrile‐0.1 M phosphate buffer (pH 7.2–7.6) as the mobile phase. Conditions included a flow rate of 1.0 mL/min, a column temperature of 35°C, a detection wavelength of 254 nm, and an injection volume of 20 μL.

### 2.8 In Vitro Antioxidant Capacity Determination

#### 2.8.1 DPPH Free Radical Scavenging Activity

Different concentrations (0.5 ~ 5.0 mg/mL) of SDP solutions were prepared. Then, 2 mL of each concentration was mixed with 2 mL of freshly prepared methanol DPPH solution (0.2 mM), and incubated in the dark at 37°C for 30 min, absorbance was measured at 517 nm. Vitamin C served as a positive control. The scavenging rate was calculated using the following formula:

$$\text{DPPH free radical scavenging rate}\;\left(\%\right)=\left[1-\left(\mathrm{Ai}-\mathrm{Aj}\right)/A0\right]\times 100\%,$$

where A0 is the absorbance of the DPPH solution, Ai is the absorbance of the sample in the DPPH solution, and Aj is the absorbance of the sample in methanol.

#### 2.8.2 ABTS Free Radical Scavenging Activity

By referring to relevant literature studies, the method for detecting the cation scavenging activity of ABTS free radicals by SDP was improved. Briefly, 1 mL SDP solution with different concentrations was absorbed and added to separate 5 mL centrifuge tubes. Subsequently, 3 mL of ABTS solution with a concentration of 7 mmol/L was added, shaken, mixed well, and allowed to react at room temperature for 20 min under light protection. The light absorption value was measured at 734 nm and recorded as A1. Subsequently, 3 mL of ultra‐pure water was used instead of ABTS solution as the control group, and the light absorption value was measured and recorded as A2. In addition, 1 mL of ultra‐pure water was used instead of the SDP solution as the blank group, and the light absorption value was determined and recorded as A0. With vitamin C as the positive control, ABTS free radical cation scavenging activity was calculated accordance with the following equation:

$$\text{ABTS}\;\text{free radical scavenging rate}\;\left(\%\right)=\left[1-\frac{\left(A1-A2\right)}{A}\right]$$

#### 2.8.3 Hydroxyl Radical Scavenging Activity

In accordance with Xie's method, scavenging activity on hydroxyl free radicals was analyzed, and several modifications were made. The SDP was prepared in accordance with the above concentration, and 2 mL of the SDP solutions with the aforementioned different concentrations were absorbed and mixed with 2 mL of FeSO_4_ solution (6 mmol/L). Then, 2 mL of hydrogen peroxide (H_2_O_2_) solution (6 mmol/L) was added to the mixed solution, and the reaction was carried out for 10 min at room temperature. After the reaction, 2 mL of salicylic acid solution (6 mmol/L) was added to continue the reaction for 0.5 h. Finally, the light absorption value of the reaction solution was detected at 510 nm. OH clearance rate was calculated in accordance with the following formula:

$$\mathrm{OH}\;\text{free radical scavenging rate}\;\left(\%\right)=\left[1-\frac{A1-A2}{A0}\right]\times 100\%$$

#### 2.8.4 Reduction Capability

The determination of the iron‐reducing antioxidant capacity of SDP was slightly modified as previously mentioned. First, 1.0 mL of Vc and SDP solution with different concentrations was accurately measured and placed into a test tube with plugs. Second, 2.5 mL of phosphate buffered saline (0.2 mol/L, pH = 6.6), and 2.5 mL of potassium ferricyanide (1%) were added, shaken well, incubated at 50°C for 20 min, and cooled rapidly. Third, 2.5 mL of trichloroacetic acid (10%) was added to stop the reaction. After the reaction was terminated, the solution was centrifuged at 5000 r/min for 10 min, 2.0 mL of the supernatant was collected, and then 2.0 mL of distilled water and 0.5 mL of 0.1% FeCl_3_ solution were added. The solution was mixed well. The reagent blank was used as reference, and absorbance at 700 nm was measured and recorded. Vitamin C was used as positive control.

### 2.9 Animal Experiment Procedure

Two‐month‐old male C57BL/6J mice (batch number 20240310) were obtained from Experimental Animal Center of Ningxia Medical University. All animal procedures were performed in accordance with the Provision and General Recommendation of Chinese Experimental Animals Administration Legislation and approved by the Ethic Committee of Ningxia Medical University (No. IACUC‐NYLAC‐2024‐193). After 1 week of adaptive feeding, mice were randomly divided into six groups (*n* = 8): control group, model group, low‐, medium‐, and high‐dose SDP groups, and a positive control group. On the first day, mice in all groups except control group were subjected to intracerebroventricular (*i.c.v*.) injection of Aβ (2 nM, 4 μL per mouse, MCE, HY‐P1363‐5), and control group mice were *i.c.v*.‐injected with normal saline (4 μL per mouse) into the lateral ventricle. On the second day, mice from control and model groups were intragastrically administered with normal saline. While the low‐, middle‐, and high‐dose SDP groups were intragastrically administered with 50, 100, or 200 mg/kg SDP, respectively, and the positive control mice were intragastrically administered with 5 mg/kg donepezil. Administration of different drugs was performed once daily for 30 days in accordance with body weight (0.2 mL/10 g), and behavioral tests including Y maze test and novel object recognition (NOR) test were performed on Days 26–30. Then the mice were sacrificed on Day 30, and hippocampal tissue was collected. Oxidative stress‐related enzymes and products including SOD, GSH‐Px, and MDA were determined using commercially available kits.

### 2.10 Behavioral Tests

#### 2.10.1 Y‐Maze Test

The Y‐maze test was conducted according to the method described in the literature to evaluate the working memory ability of AD mice (Balavigneswaran et al. 2013). The Y‐maze consists of three identical arms labeled A, B, and C for recording the movement trajectory of mice. Each arm is a quadrangular prism with a length of 40 cm, a height of 15 cm, a bottom width of 8 cm, and a top width of 12 cm. The three arms converge at an equilateral triangular center, with each arm positioned at a 120° angle relative to the others. During the experiment, the mouse is placed in the central equilateral triangle area of the Y‐maze, and its free exploration trajectory is recorded for 8 min. An entry is recorded when the mouse's entire body (excluding the tail) exits any side of the central triangle and fully enters one of the arms. Before each trial, the maze is wiped with 75% ethanol to remove any feces or debris, eliminating potential interfering cues. After 8 min, the experimental data are analyzed to calculate the spontaneous alternation rate:

$$\text{Spontaneous alternation rate}\;\left(\%\right)=\left(\text{Number of spontaneous alternations}/\left(\text{Total}\;\mathrm{arm}\;\text{entries}-2\right)\right)\times 100\%$$

For example: If the sequence of arm entries within 8 min is (BACBACBACC), the spontaneous alternations are BAC, ACB, CBA, BAC, ACB, CBA, and BAC. Thus:

$$\text{The spontaneous alternation rate}=\left[7/\left(10-2\right)\right]\times 100\%=87.5\%$$

#### 2.10.2 Novel Object Recognition Test

After 1 month of intragastric administration, the Novel Object Recognition (NOR) test was performed according to the literature to evaluate the spatial learning and memory abilities of AD mice (Duh 1998). Experimental Procedure: A clean and odor‐free testing chamber (50 cm × 50 cm × 45 cm) was used. Before and after testing, the chamber was wiped with alcohol to eliminate odors that might interfere with the mice's behavior. Noise and movement were minimized to avoid disturbing the mice during exploration. The novel object recognition test consisted of three phases: habituation, training, and testing. (1) Habituation Phase: After cleaning the chamber with alcohol, each mouse was allowed to freely explore the empty chamber for 5 min. The mouse was then returned to its home cage. (2) Training Phase: After habituation, two identical, odorless square objects (A1 and A2) were symmetrically placed in the chamber. The chamber and objects were wiped with alcohol. The mouse was placed in the center of the chamber and allowed to explore the objects for 5 min. After exploration, the mouse rested for 5 min. (3) Testing Phase: After the training phase, object A2 was replaced with a novel object B (different in size, shape, and color). The chamber and objects were wiped with alcohol. The mouse was placed in the center and allowed to explore the objects for 5 min. The number of times the mouse explored each object was recorded. Exploration Criteria: Exploration was defined as when the mouse's head was oriented toward the object, or when it touched or sniffed the object.

The Preference Index (PI) was calculated for each mouse using the following formula:

$$\mathrm{PI}=\mathrm{tB}/\left(\mathrm{tA}1+\mathrm{tB}\right)\times 100\%$$

PI: Preference Index; tB: Exploration times of the novel object (B); tA1: Exploration times of the familiar object (A1); The PI reflects the mouse's ability to recognize and prefer the novel object, indicating memory retention.

### 2.11 Western Blot Analyses

Total protein from whole mouse brain was extracted and analyzed via Western blot analysis. Protein concentration was determined using a bicinchoninic acid (BCA) kit. Equal amounts of protein (30–40 μg) were separated on 10% sodium dodecyl sulfate–polyacrylamide gel electrophoresis gel and transferred to a polyvinylidene fluoride membrane. The membrane was blocked and incubated overnight with primary antibodies against Kelch‐like ECH‐associated protein 1 (Keap1), nuclear factor erythroid 2‐related factor 2 (Nrf2), and quinone oxidoreductase 1 (NQO1) at 4°C. After washing, secondary antibodies were added, and protein bands were visualized using an electrochemiluminescence kit. Bands were quantified using Image J software and normalized to β‐actin.

### 2.12 Statistical Analyses

All statistical analyses were conducted with the Prism graphical software 8.3.0 (GraphPad software, San Diego, California, USA). Data were generated from multiple repeats of different biological experiments to obtain the mean values and SEM displayed throughout. Comparisons between multiple groups were made by one‐way ANOVA followed by Tukey's post hoc test. *p* < 0.05 was considered statistically significant.

## 3 Results and Discussion

### 3.1 Ultraviolet Analysis and Infrared Analysis

**FIGURE 1:**
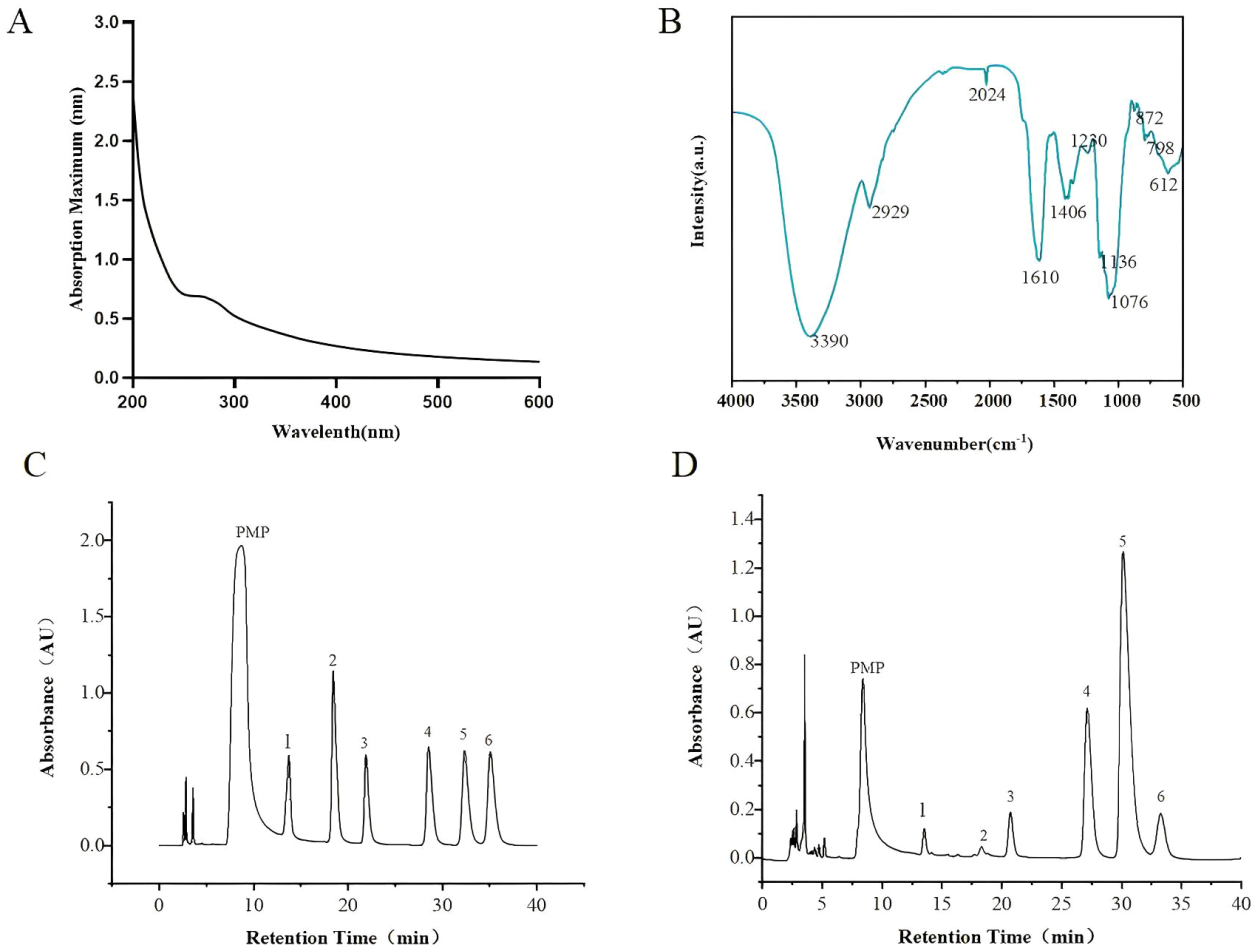
Spectral and chromatographic analysis results of SDP. (A) Ultraviolet spectrum of SDP. (B) FT‐IR spectrum of SDP. (C) HPLC analysis of monosaccharide composition in the mixed standard solution (1: D‐Man, 2: L‐Rha, 3: D‐Gal, 4: D‐Glu, 5: D‐Gala, 6: Ara). (D) HPLC analysis of monosaccharide composition of SDP (1: D‐Man, 2: L‐Rha, 3: D‐Gal, 4: D‐Glu, 5: D‐Gala, 6: Ara).

As shown in Figure 1A, there are absorption peaks at wavelengths of 260 nm and 280 nm (Okamoto and Okabe 2000), indicating that the SDP solution contains a small amount of nucleic acid and protein.

The infrared spectrum is commonly used to determine functional groups of polysaccharides, and the structure of SDP is identified and analyzed in accordance with the characteristic absorption peak displayed by an infrared spectrum. The infrared spectrum of the SDP is shown in Figure 1B. The analysis of the figures indicates that the SDP exhibits a strong and wide absorption peak within the range of 3500 and 3300 cm^−1^, which is caused by the ‐OH stretching vibration of the associated SDP molecules (Long et al. 2022). Absorption peaks within the range of 2930–2900 cm^−1^ suggest the presence of H—C=O groups and C—H stretching vibration peaks in CH_2_ (Wen et al. 2017). The absorption peak near 1400 cm^−1^ before and after purification indicates that the sugar chain contains carboxyl ‐COOH, further showing that the SDP contains uronic acid (Zhong et al. 2021). The peak near 1600 cm^−1^ is the absorption peak of the symmetric deformation vibration of the carbonyl group, indicating that the polysaccharide contains carbonyl group before and after purification. Before and after purification, SDP exhibits strong absorption peaks near 1100–1000 cm^−1^, which is the characteristic region of the pyranosaccharide main chain (Hashemifesharaki et al. 2020). The weak absorption peak near 800 cm^−1^ indicates that the polysaccharide contains β‐type glycoside. The absorption peak near 600 cm^−1^ indicates that the polysaccharide contains α‐type glycoside (Dong et al. 2015).

### 3.2 SEM Analysis

**FIGURE 2:**
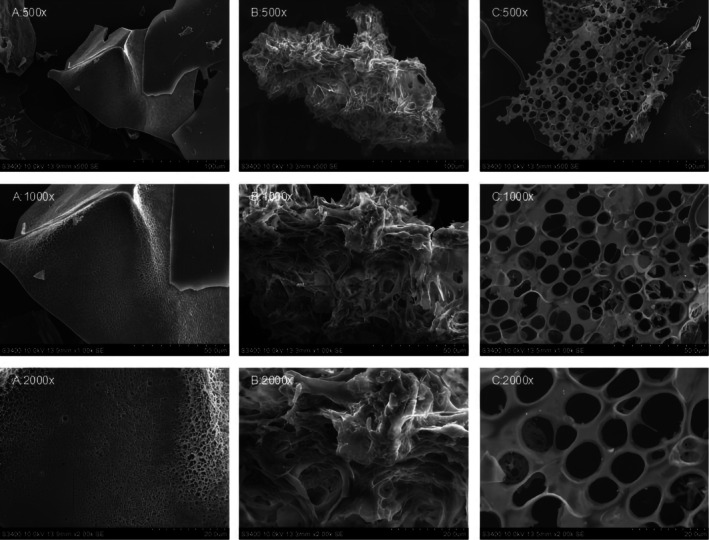
Different morphologies of SDP observed with SEM. (A) SDP exists in the form of sheets with small pores. (B) SDP exists in bulk form. (C) SDP exists in the form of sheets with large pores.

The spatial structure of SDP can affect structure–activity relationship. SEM was used to identify the spatial morphology of SDP, which was shown in Figure 2 by magnifying 500×, 1000×, and 2000×, respectively. SDP mainly exists in three different forms: the form of sheets with small pores (Figure 2A), the bulk form (Figure 2B), and the form of sheets with large pores (Figure 2C).

### 3.3 NMR Analysis

NMR spectroscopy is one of the commonly used techniques for the structural analysis of complex polysaccharides, simplifying the structural analysis of carbohydrates (Chen et al. 2021). ^1^H‐NMR is primarily used to analyze glycosidic bond configurations in polysaccharide structures. In the ^1^H‐NMR spectrum, peaks within the range of δ 3.0–5.5 are typical for polysaccharides (Guo et al. 2019). The chemical shifts of polysaccharides are mostly concentrated within the range of δ 3.5–4.4 ppm, while the region of δ 4.4–5.8 ppm corresponds to the anomeric proton region. In generally, the anomeric hydrogen of α‐configured pyranose residues has a δ value higher than 5.0 ppm, while that of β‐configured pyranose residues has a δ value lower than 5.0 ppm. The anomeric hydrogen of furanose rings typically appears at around δ 5.4 ppm (Xia et al. 2025). The chemical shift range of ^13^C‐NMR is broader than that of ^1^H‐NMR. The resonance signals of anomeric carbons usually appear within the range of δ 90–112 ppm. Typically, α‐configured pyranose residues fall within δ 90–102 ppm, while β‐configured pyranose residues are found within the range of δ 102–112 ppm (Jiang et al. 2024). The C3 or C5 carbons of pyranose rings generally appear below δ 80 ppm, and the characteristic peaks of the pyranose ring are observed within the range of δ 72–80 ppm. The ^1^H‐NMR spectrum of SDP (Figure S1) shows that the chemical shifts of anomeric hydrogen protons are within the range of δ 4.0–5.54 ppm, exhibiting typical polysaccharide characteristic peaks. This finding indicates that SDP contains β‐configured and α‐configured pyranose residues. The ^13^C‐NMR spectrum of SDP (Figure S2) reveals a carbon signal at δ 98.73 ppm, indicating the presence of α‐configured pyranose residues, and a carbon signal at δ 103.5 ppm, indicating the presence of β‐configured pyranose residues.

### 3.4 Determination of Monosaccharide Components

The monosaccharide composition of SDP was qualitatively analyzed using pre‐column derivatization PMP‐HPLC (Xu et al. 2016). The HPLC chromatogram of SDP is presented in Figure 1C,D. Through this analysis, we have identified six monosaccharide components in SDP: galactose, glucose, arabinose, galacturonic acid, mannose, and rhamnose. Furthermore, the molar ratios of these monosaccharides have been determined to be as follows: galactose: glucose: arabinose: galacturonic acid: mannose: rhamnose = 5.561: 2.224: 0.802: 0.616: 0.613: 0.184. This detailed composition analysis provides valuable insights into the structural characteristics and functional properties of SDP, contributing to a deeper understanding of its biological activities and potential applications.

### 3.5 In Vitro Antioxidant Capacity Determination

**FIGURE 3:**
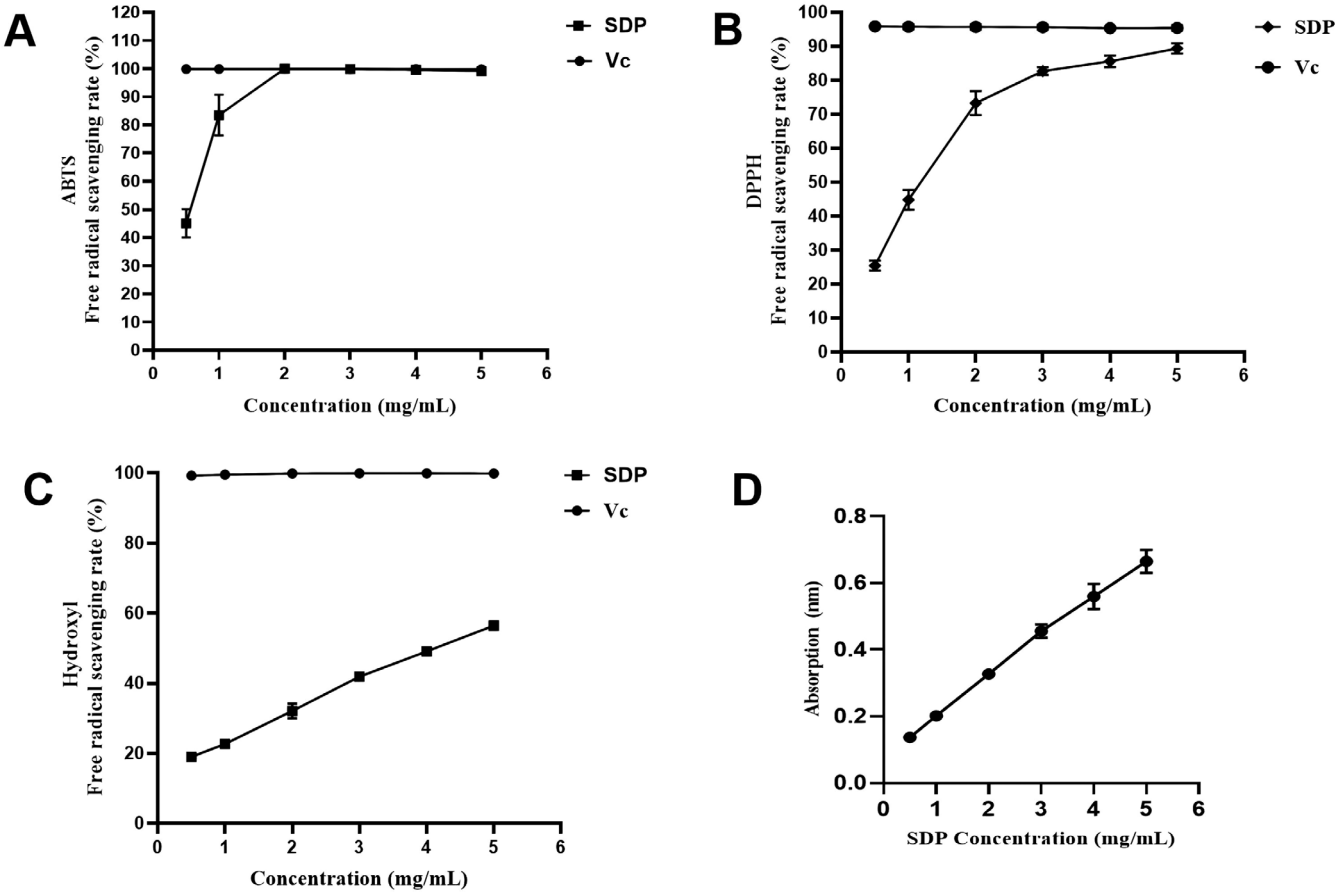
SDP exerts antioxidant activity in vitro. (A) Effects of SDP and vitamin C (Vc) on the ABTS radical scavenging rate. (B) Effects of SDP and Vc on the DPPH radical scavenging rate. (C) Effects of SDP and Vc on the hydroxyl radical scavenging rate. (D) The reducing power of SDP.

ABTS free radicals exhibit strong absorption at 734 nm, and compounds with hydrogen donors can clear ABTS free radicals, weaken their color, and reduce their absorbance (Ghavi 2015). As shown in Figure 3A, the SDP demonstrated strong ABTS free radical scavenging activity, with the average scavenging rates reaching 99.07% at the concentration of 5 mg/mL. Within the concentration range of 0.5 ~ 2.0 mg/mL, the ABTS free radical scavenging ability of the SDP increased with an increase in concentration. When the concentration was higher than 2 mg/mL, the scavenging ability of the SDP tended to be stable and was close to that of the Vc with the same concentration. The results showed that SDP exhibited potent ABTS scavenging activity.

The DPPH radical is a stable free radical with maximum absorption at 517 nm and it is easily eliminated by antioxidants. Therefore, it has been widely accepted as a tool for evaluating the free radical scavenging activity of natural compounds (Luo and Fan 2011). As shown in Figure 3B, the SDP demonstrated certain DPPH free radical scavenging ability, with the average scavenging rates reaching 89.44% at the concentration of 5 mg/mL. Within the experimental concentration range (0.5–5.0 mg/mL), the DPPH clearance rate of the SDP and Vc exhibited a gradually increasing trend with increased concentration.

Hydroxyl free radicals, reactive oxygen species produced by oxidative metabolism in organisms, cause oxidative damage to carbohydrates, proteins, DNA and lipids by interacting with endogenous biomolecules (Yuan et al. 2008; Balavigneswaran et al. 2013), leading to cellular apoptosis and the development of multiple diseases. Therefore, hydroxyl radical clearance is also one of the indexes for evaluating the antioxidant activity of antioxidants. As shown in Figure 3C, SDP exhibited certain hydroxyl radical scavenging activity, with the average scavenging rates reaching 56.43% at the concentration of 5 mg/mL. Within the 0.5–2.0 mg/mL concentration range, the hydroxyl radical scavenging ability of the SDP increased with an increase in concentration.

Reducing power is a key indicator for evaluating the antioxidant activity of antioxidants. Its assay principle is based on the reduction of potassium ferricyanide to potassium ferrocyanide by the antioxidant. The resulting potassium ferrocyanide then reacts with ferric chloride to form a blue‐colored complex, which has a maximum absorption peak at a wavelength of 700 nm. A higher absorbance value indicates a greater reduction of potassium ferricyanide, and a stronger reducing power of the antioxidant corresponds to more potent antioxidant activity (Duh 1998). As shown in Figure 3D, SDP exhibited a reducing capacity comparable to that of Vc, the positive control. Within the concentration range of 0.5–5.0 mg/mL, the reducing ability of SDP increased in a concentration‐dependent manner.

### 3.6 Results of Behavioral Test

**FIGURE 4:**
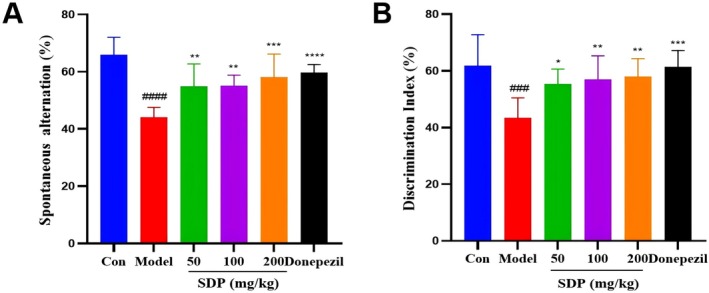
Effect of SDP on cognitive dysfunction in Aβ‐induced AD mice. (A) Effect of SDP on working memory function evaluated by Y‐maze test. (B) Effect of SDP on discriminative ability for novel objects evaluated by novel object recognition (NOR) test. Compare with control group, ####*p* < 0.0001, ###*p* < 0.001; compare with model group, *p < 0.05, **p < 0.01, ***p < 0.001, ****p < 0.0001. Data are presented as mean ± SEM (*n* = 8).

The Y‐maze test was used to examine the effect of SDP on the working memory ability of Aβ‐induced AD mice. As shown in Figure 4A, compared with the control group, the spontaneous alternation rate of mice in the model group decreased, suggesting that Aβ could impair the working memory ability of mice. In comparison with the model group, treatment with SDP increased the spontaneous alternation rate, indicating that SDP can ameliorate Aβ‐induced working memory impairment.

The NOR test was conducted to evaluate the effect of SDP on the ability of novel object recognition in Aβ‐induced AD mice. As shown in Figure 4B, compared with the control group, the novel object discrimination index of the model group mice decreased, indicating Aβ‐impaired novel object recognition ability. In contrast, the SDP‐treated mice showed an increased discrimination index compared to the model mice, suggesting that SDP can alleviate Aβ‐induced impairment of novel object recognition in AD mice.

### 3.7 In Vivo Anti‐Oxidative Stress Effect of SDP

**FIGURE 5:**
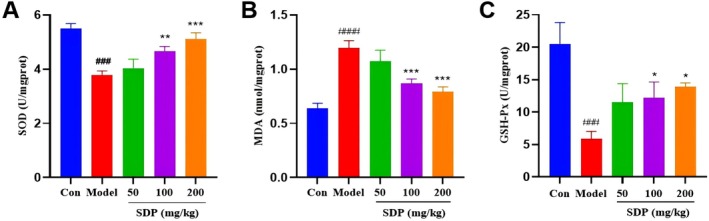
Effects of SDP on the levels of oxidative stress‐related enzymes and products in the hippocampus of AD mice. (A) Effect of SDP on SOD contents. (B) Effect of SDP on MDA contents. (C) Effect of SDP on GSH‐Px contents. Compared with the control group, ####*p* < 0.0001, ###*p* < 0.001; compared with the model group, *p < 0.05, **p < 0.01, ***p < 0.001. Data are presented as mean ± SEM (*n* = 3).

Oxidative stress refers to an imbalance state that is formed when the production of oxygen free radicals exceeds the scavenging capacity of antioxidants. In the development process of AD, Aβ is closely related to oxidative stress (Chen and Zhong 2014). Aβ can generate peroxides and free radicals through various pathways, exacerbating peroxidative damage to neuronal cells (Forman and Zhang 2021). In AD, Aβ deposited in the brain can promote the release of reactive oxygen species such as malondialdehyde (MDA) and H_2_O_2_, leading to a decrease in mitochondrial membrane potential, imbalance in metal homeostasis, and synaptic dysfunction, ultimately resulting in cognitive impairment. Antioxidant enzymes such as superoxide dismutase (SOD), NQO1, and heme oxygenase‐1 can scavenge these pro‐oxidative factors (Chen and Zhong 2014). The experimental results presented in Figure 5 indicate that the levels of SOD and glutathione peroxidase (GSH‐Px) in the hippocampal region of the model group mice were significantly reduced compared with those of the control group mice, while the level of MDA was significantly increased. This finding suggests that Aβ induction leads to increased free radical attacks in mouse brain, weakening the ability to scavenge free radicals and promoting oxidative stress. Compared with that of the model group, the medium and high doses of SDP significantly increased the levels of SOD and GSH‐Px while significantly decreasing the level of MDA. By contrast, the low dose of SDP had no significant effect on the levels of SOD, GSH‐Px, and MDA in mouse brain. This finding indicates that SDP can protect mouse brain from free radical attacks. Moreover, its ability to scavenge free radicals increases with dosage, thereby preventing oxidative stress in mouse brain.

Oxidative stress is considered one of the pathogenic mechanisms of AD, and it plays a significant role in the onset and progression of this disease. Nrf2 is a key regulator of oxidative stress defense. Under physiological conditions, Nrf2 binds to its cytoplasmic chaperone Keap1, and Nrf2 activity is relatively suppressed in the cytoplasm. Under oxidative stress conditions, the Nrf2‐Keap1 interaction is disrupted in a dose‐dependent manner. Free and newly synthesized Nrf2 translocates to the nucleus and heterodimerizes with a small Maf protein (Qu et al. 2020). Keap1 is a negative regulatory protein that is capable of sensing cellular oxidative stress and precisely modulating Nrf2 activity. Under oxidative stress, oxidative signals directly induce conformational changes in the Keap1 domain, leading to increased Nrf2 activity. Nrf2 then translocates to the nucleus and binds to the antioxidant response element (ARE) (Quinti et al. 2017), initiating the Keap1/Nrf2/ARE antioxidant pathway. NQO1 is a ubiquitous flavoprotein in eukaryotic cells that specifically catalyzes intracellular two‐electron reduction reactions, detoxifying quinones and protecting cells. As an antioxidant enzyme, the physiological activation of NQO1 can effectively prevent neuropathological damage (Jamuna et al. 2018).

**FIGURE 6:**
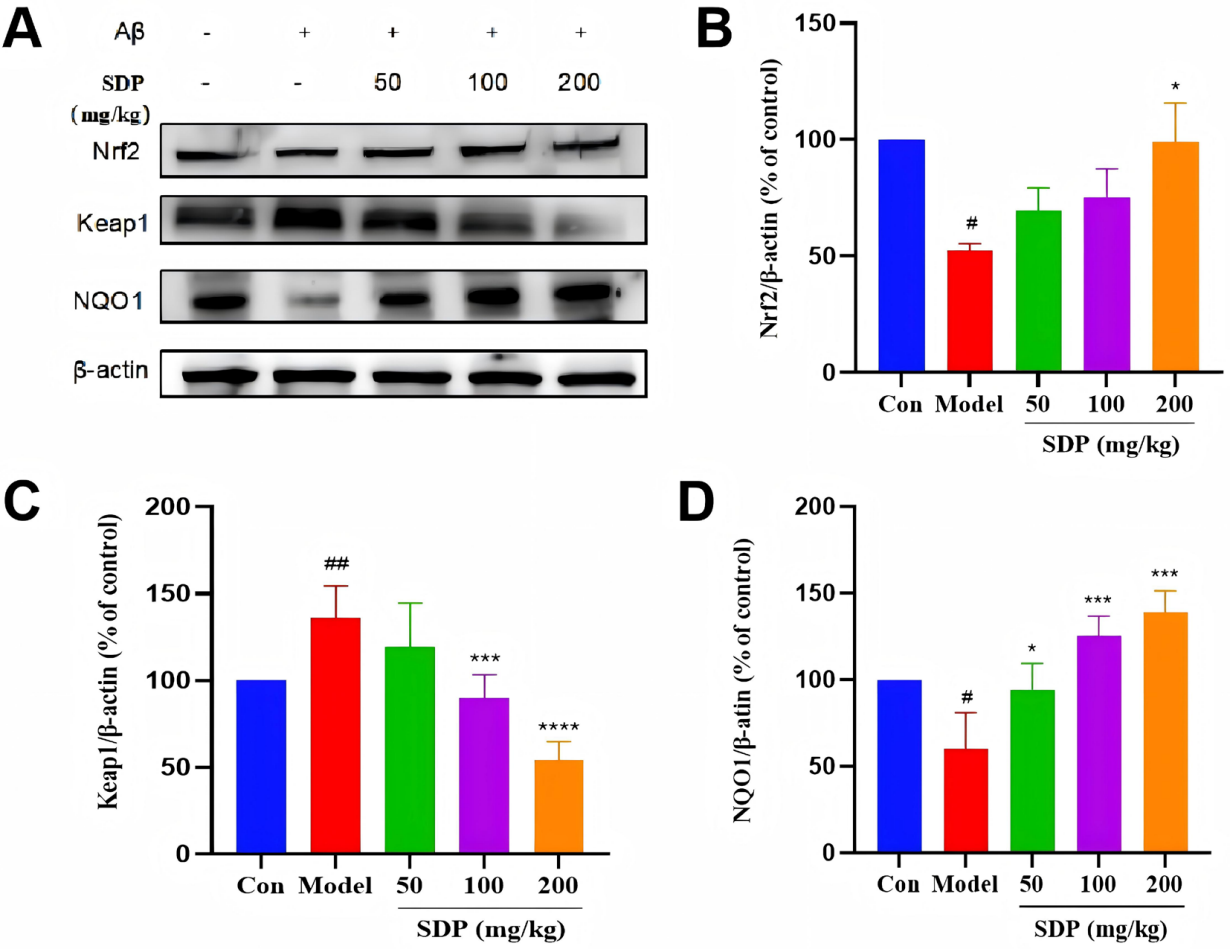
Effect of SDP on the expression of oxidative stress‐related proteins in the brains of Aβ‐induced AD mice. (A) Western blotting results of the effect of SDP on the expression of Nrf2, Keap1 and NQO1. (B) Quantitative analysis of Nrf2 protein expression. (C) Quantitative analysis of Keap1 protein expression. (D) Quantitative analysis of NQO1 protein expression. Compared with the control group, #*p* < 0.05, ##*p* < 0.01; compared with the model group, *p < 0.05, ***p < 0.001, ****p < 0.0001. Data are presented as mean ± SEM (*n* = 3).

We utilized Western blot experiments to investigate whether SDP ameliorates oxidative stress in AD mice through the Keap1/Nrf2/NQO1 pathway. The results (Figure 6) showed that the expression levels of Nrf2 and NQO1 proteins in the brains of the model group mice were significantly reduced compared with those of the control group mice, while the expression of Keap1 protein was significantly increased. This finding suggests that Aβ induction suppresses key regulatory proteins of oxidative stress in the brains of AD mice, leading to an imbalance in oxidative stress levels. Compared with those of the model group, the expression levels of Nrf2 and NQO1 proteins in the brains of the SDP treatment group were significantly increased, while the expression of Keap1 protein was significantly decreased. This result indicates that the imbalance in oxidative stress levels in the brains of AD mice is ameliorated after treatment with SDP, further demonstrating that the therapeutic effect of SDP on AD mice may be mediated through the Keap1/Nrf2/NQO1 pathway, alleviating learning and memory impairments in mice.

### 3.8 Structure–Activity Relationship Between SDP Structural Features and Antioxidant Activity

The biological activity of polysaccharides is closely related to their structural characteristics, including purity, monosaccharide composition, and morphology (Zhou et al. 2021). The total saccharide content of purified SDP in this study was determined to be 86.37% via the phenol‐sulfuric acid method, with only small amounts of nucleic acids and proteins detected by UV spectroscopy. High polysaccharide purity is a prerequisite for exerting stable biological activity. Impurities such as proteins can form complexes with polysaccharides through hydrogen bonds or hydrophobic interactions, thereby hindering the binding of polysaccharides to free radicals and reducing antioxidant activity (Wang et al. 2025). The low impurity content of SDP ensures its potent scavenging effects on ABTS, DPPH, and hydroxyl radicals in vitro.

Our results reveal that SDP is composed of galactose, glucose, arabinose, galacturonic acid, mannose, and rhamnose. Among them, galactose accounts for the highest proportion, followed by glucose. It has been reported that polysaccharides rich in galactose and glucose usually exhibit strong antioxidant activity (Zhang et al. 2024). The presence of uronic acid endows SDP with a negative charge, which can chelate metal ions (such as Fe^2+^) and inhibit the Fenton reaction, thereby reducing the production of hydroxyl radicals (Zhong et al. 2021). NMR analysis revealed that SDP contains both α‐ and β‐type glycosidic bonds. The β‐glycosidic bond is more stable and not easily hydrolyzed, which helps SDP maintain its structural integrity in vitro and in vivo (Chen et al. 2021).

The SEM results showed that SDP exists in three forms: sheets with small pores, bulk form, and sheets with large pores. The porous structure increases the specific surface area of SDP, which enhances the contact probability between SDP and free radicals, thereby improving the free radical scavenging efficiency (Hashemifesharaki et al. 2020). In addition, the sheet structure of SDP is more conducive to penetration into cells and exerting intracellular antioxidant effects, such as reducing MDA levels and increasing SOD and GSH‐Px activities in the hippocampus of AD mice.

## 4 Conclusion

This study characterized the structural characteristics of SDP via UV, FT‐IR, NMR, HPLC, and SEM, revealing a polysaccharide composed of mannose, rhamnose, galacturonic acid, glucose, galactose, and arabinose. In vitro tests demonstrated SDP's potent antioxidant activity against ABTS, DPPH, and hydroxyl radicals in a dose‐dependent manner, with the average scavenging rates reaching 99.07%, 89.44%, and 56.43% respectively at the concentration of 5 mg/mL. In vivo, SDP ameliorated cognitive dysfunction in AD mice, relieved oxidative stress, and activated the Nrf2/Keap1/NQO1 pathway, suggesting therapeutic potential via antioxidant mechanisms.

Despite the significant findings of this study, several limitations need to be acknowledged. The fine structure of SDP, such as glycosidic bond linkage sites and chain branching degree, was not fully characterized. The anti‐AD mechanism was only explored via the Keap1/Nrf2/NQO1 pathway, with other potential pathways like Aβ clearance and neuroinflammation regulation remaining uninvestigated. For future research, advanced structural analysis techniques such as high‐performance gel permeation chromatography (HPGPC) and atomic force microscopy (AFM) could be employed to clarify the fine structure of SDP. Second, multi‐omics technologies including transcriptomics, proteomics, and metabolomics can be used to comprehensively explore the molecular mechanisms underlying the anti‐AD effect of SDP. These studies will facilitate the development of SDP as a potential therapeutic agent for AD.

## Author Contributions

**Meixia Sun:** investigation, formal analysis, and writing – original draft. **Jiaxin He:** investigation, methodology, and validation. **Huan Song:** methodology, validation, and data curation. **Zhi Ma:** methodology and validation. **Xilian Zhang:** formal analysis. **Yao Yao:** conceptualization, supervision, and writing – review and editing. **Juan Li:** conceptualization, writing – review and editing, funding acquisition, and supervision.

## Funding

This work was supported by the Key R&D Program of Ningxia (2024BEG01006), National Natural Science Foundation of China (82160759, 82060729), and Natural Science Foundation of Ningxia (2024AAC02049, 2024AAC03294).

## Conflicts of Interest

The authors declare no conflicts of interest.

## Supporting information


**Figure S1:** ^1^H‐NMR spectrum of SDP.
**Figure S2:** ^13^C‐NMR spectrum of SDP.

## Data Availability

The original contributions presented in the study are included in the article; further inquiries can be directed to the corresponding author.
