# Supplementary figures and images for: Structural Characterization and Anti‐Alzheimer's Disease Effect of Polysaccharides From Stellariae Radix
Source: Food Sci Nutr. 2026 Mar 11;14(3):e71604. doi: 10.1002/fsn3.71604 (PMC13093678; doi:10.1002/fsn3.71604)

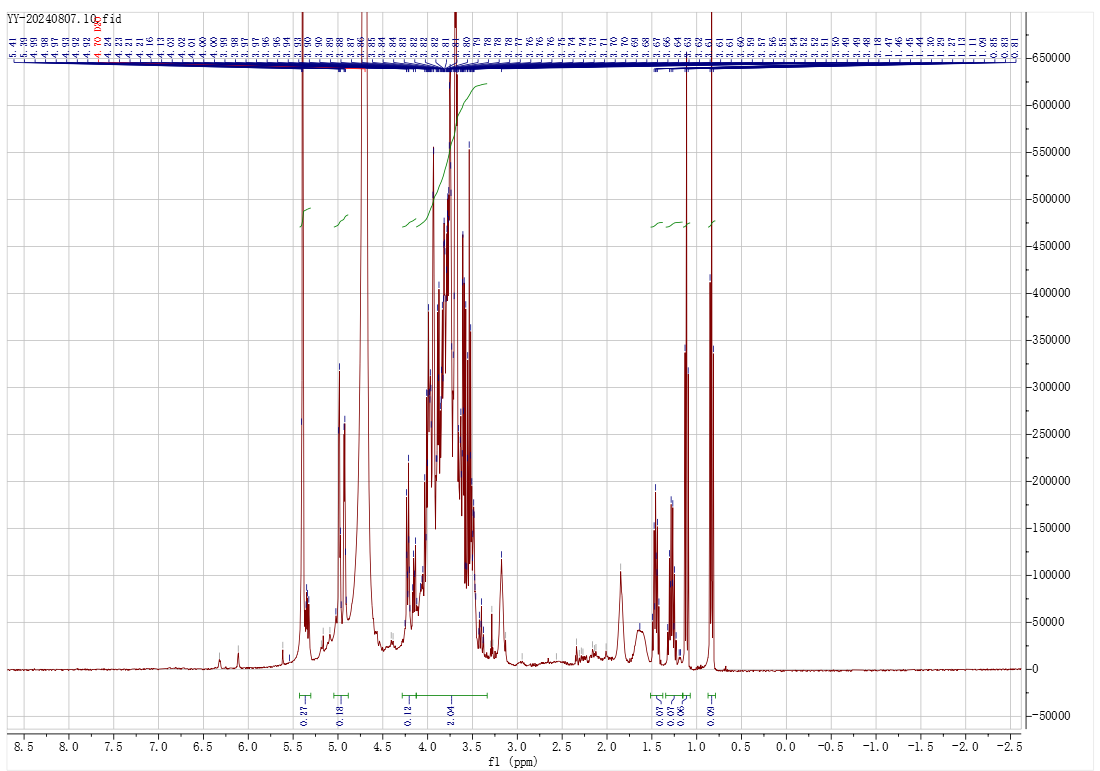
Fig S1. 1H-NMR Spectrum of SDP


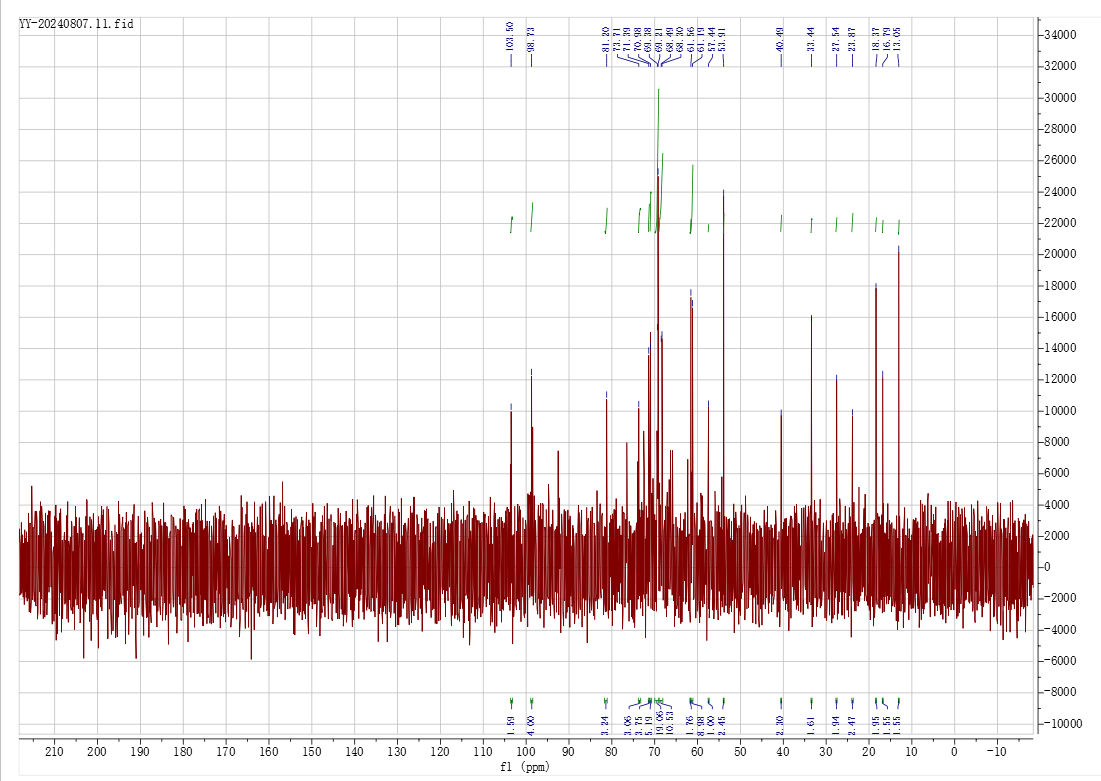
Fig S2. 13C-NMR Spectrum of SDP

Supplement: Supplementary file 1 — Figure S1: 1H‐NMR spectrum of SDP. Figure S2: 13C‐NMR spectrum of SDP. [file FSN3-14-e71604-s001.docx]
